# Supplementary material for: Let-7a Downregulation Accompanied by KRAS Mutation Is Predictive of Lung Cancer Onset in Cigarette Smoke–Exposed Mice
Source: Int J Mol Sci. 2023 Jul 21;24(14):11778. doi: 10.3390/ijms241411778 (PMC10380304; doi:10.3390/ijms241411778)
Supplement: Supplementary file 1 [file ijms-24-11778-s001.zip › ijms-2445830-supplementary.pdf]

LET-7a-1 MOUSE PROMOTER

(da http://www.nature.com/cmi/journal/v11/n1/full/cmi201351a.html)

Genomic sequence

chr13:48,538,171-48,539,472

TGGTAATCAGCCCCCAACCTGAAACTGGGCCTCTGGGCACGGGGGATTTAGATATACTATTTCAGGATCACTAACTTGAGTCC
AATACTAACTTAAACTTGTATTTGATGATGGCTCAAATTCTTAGTGAATTATTAAACTTAAAAGTAGCTTTGCCTTTTCACTA
TTCTCAACAACAGTCTATGAGATTCTAGTTTGTGTACATCCTTGCTAGAGCTTACTAATATCTTAGAAATGTTTGTGTGTGGT
AGTCAGAGGATGACTTGTGAAAATCAGCTCTCCTCACCATGTGGATCCTGAAGATTGAATTTA**C/TG**CTTTCAGGCTTGG**C/T**
GATAGGCAACCTTATTCCCTGAATATATATCTCTAGTCCTCATTACCTTTTTGATGGATAGCTTTAATGGATGTGTGTTGTC
CTGTTTTGGATAGGTATATTCATAATGACTAAAGAATGATAATTTAGATCTTTCCTGTACTTATTGGGTATTTGTGTTTATGA
GCCATATCGTCTCTGCTTCTAGATCAGTAGCTGTCTCAGCTCTGGTAAATACCTCTTGCCTGGTTTTTGTTTTTTGAGATTGG
GTTTTAATTTTGTAGCTTGGTTTGGCCTCAAATTTACAGCAGTCCTCTTGTCCCAACCTCCCTGGTGCTCTTAATGAAAATCT
GTTGACCTTGAATATGGCTTTATTTTTTGAACTGTAACTTACATGACATTGAATCAATATGTTCTTGCTTATCTTTGTAATCT
TTACTATTAGGTCACTACATAATTCATTTGTCTCATGAACATCTTTTCCTACTCTTTGATAATTTCACGTATATTTAATGTGC
CCATAATTTTGGCTACGATATCACACACCAGCTATAAACTTTCATTTGGTCATTTCATACTGGTACTCTGGGAACTAGGTTTT
TGGATTGGGGACATTTAAGCTTCAGTATTAGACAGTGGTAAGAATGTAATTTCTAGATCTTATAACATATTGAATTAGAGGCT
TATAGCCCAGGTGTATCATATAATACAATGAAACTACAATATAGGTCATATATGTAACTGTTCAGTTAAACTTCATGTTCAAG
TTAAAGTAGTGAAAATCTAATATTTTCTTTTCCCTCATTACACAGGAAACCGGAATTACAAAGGAGAACGGCTTCCTGTGATG
CTCAGCTGTGATTACTTTCAACATTCACCCTGGATGTTCTCTT**CACTGTGGGATGAGGTAGTAGGTTGTATAGTTTTAGGGTC**
ACACCCACCACTGGGAGATAACTATACAATCTACTGTCTTTCCTAAGGTGATGGAAAAGT

Bisulfite converted sequence

TGGTAATTAGTTTTTAATTTGAAATTGGGTTTTTGGGTA**C/TG**GGGGATTTAGATATATTATTTTAGGATTATTAATTTGAGT
TTAATATTAATTTAAATTTGTATTTGATGATGGTTTAAATTTTTAGTGAATTATTAAATTTAAAAGTAGTTTTGTTTTTTTAT
TATTTTTAATAATAGTTTATGAGATTTTAGTTTGTGTATATTTTTGTTAGAGTTTATTAATATTTTAGAAATGTTTGTGTGTG
GTAGTTAGAGGATGATTTGTGAAAATTAGTTTTTTTTATTATGTGGATTTTGAAGATTGAATTTA**C/TG**TTTTTTAGGTTTGG
C/TGATAGGTAATTTTATTTTTTGAATATATATTTTTAGTTTTTATTATTTTTTTTGATGGATAGTTTTAATGGATGTGTGTT
GTTTTGTTTTGGATAGGTATATTTATAATGATTAAAGAATGATAATTTAGATTTTTTTTGTATTTATTGGGTATTTGTGTTTA
TGAGTTATAT**C/TG**TTTTGTTTTTAGATTAGTAGTTGTTTTAGTTTTGGTAAATATTTTTTGTTTGGTTTTTGTTTTTTGAG
ATTGGGTTTTAATTTTGTAGTTTGGTTTGGTTTTAAATTTATAGTAGTTTTTTTGTTTTAATTTTTTTGGTGTTTTTAATGAA
AATTTGTTGATTTTGAATATGGTTTTATTTTTTGAATTGTAATTTATATGATATTGAATTAATATGTTTTTGTTTATTTTTTGT
AATTTTTATTATTAGGTTATTATATAATTTATTTGTTTTATGAATATTTTTTTTTTATTTTTTGATAATTTTA**C/TG**TATATTT
AATGTGTTTATAATTTTGGTTA**C/TG**ATATTATATATTAGTTATAAATTTTTATTTGGTTATTTTATATTGGTATTTTGGGAA
TTAGGTTTTTGGATTGGGGATATTTAAGTTTTAGTATTAGATAGTGGTAAGAATGTAATTTTTAGATTTTATAATATATTGAA
TTAGAGGTTTATAGTTTAGGTGTATTATATAATATAATGAAATTATAATATAGGTTATATATGTAATTGTTTAGTTAAATTTT
ATGTTTAAGTTAAAGTAGTGAAAATTTAATATTTTTTTTTTTTTTATTATATAGGAAAT**C/TG**GAATTATAAAGGAGAA**C/TG**
GTTTTTTGTGATGTTTAGTTGTGATTATTTTTAATATTTATTTTGGATGTTTTTTT**TATTGTGGGATGAGGTAGTAGGTTGTA**
TAGTTTTAGGGTTATATTTATTATTGGGAGATAATTATATAATTTATTGTTTTTTTTAAGGTGATGGAAAAGT

Pcr primers

Let7 F1: TGGTAGTTAGAGGATGATTTGTGA

Let7 RV1: Bio- TATCCAAAACAAAACAACACACAT

PCR: 179bp

Sequencing primer:

Let7 Seq1: GGATTTTGAAGATTGAATT

PCR: 179bp

Temperature	Time	Ciclyes
95°C	5’	1
95°C	30”	45
52°C	30”	
72°C	30”	
72°C	5’	1
6°C	∞	1

**Reagents**	**Volume (µl)**
**H2O**	13
**HotStart Mix**	25
**Fw Primer**	1
**Rv Primer R**	1
**DNA**	10
